# A novel mutation of *SATB2* inhibits odontogenesis of human dental pulp stem cells through Wnt/*β*-catenin signaling pathway

**DOI:** 10.1186/s13287-021-02660-8

**Published:** 2021-12-04

**Authors:** Tianyi Xin, Qian Li, Rushui Bai, Ting Zhang, Yanheng Zhou, Yuehua Zhang, Bing Han, Ruili Yang

**Affiliations:** 1 Department of Orthodontics, Peking University School and Hospital of Stomatology & National Center of Stomatology & National Clinical Research Center for Oral Diseases & National Engineering Laboratory for Digital and Material Technology of Stomatology & Beijing Key Laboratory of Digital Stomatology & Research Center of Engineering and Technology for Computerized Dentistry Ministry of Health & NMPA Key Laboratory for Dental Materials, No. 22 Zhongguancun South Avenue, Haidian District, Beijing, 100081 People’s Republic of China; 2 Department of Pediatrics, Peking University First Hospital, Beijing, 100034 People’s Republic of China

**Keywords:** SATB2, Tooth agenesis, Odontogenesis, Dental pulp stem cells, Wnt/*β*-catenin signaling

## Abstract

**Background:**

*SATB2*-associated syndrome (SAS) is a multisystem disorder caused by mutation of human *SATB2* gene. Tooth agenesis is one of the most common phenotypes observed in SAS. Our study aimed at identifying novel variant of SATB2 in a patient with SAS, and to investigate the cellular and molecular mechanism of tooth agenesis caused by *SATB2* mutation.

**Methods:**

We applied whole exome sequencing (WES) to identify the novel mutation of *SATB2* in a Chinese patient with SAS. Construction and overexpression of wild-type and the mutant vector was performed, followed by functional analysis including flow cytometry assay, fluorescent immunocytochemistry, western blot, quantitative real-time PCR and Alizarin Red S staining to investigate its impact on hDPSCs and the underlying mechanisms.

**Results:**

As a result, we identified a novel frameshift mutation of *SATB2* (c. 376_378delinsTT) in a patient with SAS exhibiting tooth agenesis. Human DPSCs transfected with mutant SATB2 showed decreased cell proliferation and odontogenic differentiation capacity compared with hDPSCs transfected with wild-type SATB2 plasmid. Mechanistically, mutant SATB2 failed to translocate into nucleus and distributed in the cytoplasm, failing to activate Wnt/*β*-catenin signaling pathway, whereas the wild-type SATB2 translocated into the nucleus and upregulated the expression of active* β*-catenin. When we used Wnt inhibitor XAV939 to treat hDPSCs transfected with wild-type SATB2 plasmid, the increased odontogenic differentiation capacity was attenuated. Furthermore, we found that SATB2 mutation resulted in the upregulation of DKK1 and histone demethylase JHDM1D to inhibit Wnt/*β*-catenin signaling pathway.

**Conclusion:**

We identified a novel frameshift mutation of SATB2 (c.376_378delinsTT, p.Leu126SerfsX6) in a Chinese patient with *SATB2*-associated syndrome (SAS) exhibiting tooth agenesis. Mechanistically, SATB2 regulated osteo/odontogenesis of human dental pulp stem cells through Wnt/*β*-catenin signaling pathway by regulating DKK1 and histone demethylase JHDM1D.

**Supplementary Information:**

The online version contains supplementary material available at 10.1186/s13287-021-02660-8.

## Background

*SATB2*-associated syndrome (SAS) is a recently named multisystem disorder manifesting as developmental delay with limited speech, craniofacial abnormalities including dental anomalies and cleft palate, facial dysmorphism and behavioral problems [1, 2]. SAS is caused by mutations of *SATB2* including single nucleotide alterations, deletions and insertions, duplications and translocations [2, 3]. Special AT-rich sequence binding protein 2 (SATB2) is a transcription factor and regulates multiple developmental processes [4–6]. In mice, loss of *Satb2* results in cleft palate, shorter lower jaw and missing of incisor teeth [5]. These manifestations are consistent with the phenotype in human with *SATB2* mutation [3, 7–9]. In a research comprised of 72 patients with SAS, dental anomalies were present in all individuals after one year old, which include hypodontia, delayed eruption, larger incisors and malformed teeth [8, 10]. It has been proved that SATB2 plays a crucial role in skeletal development and regulates several osteogenic-related genes such as *Runx2* and *Bsp* [5]. However, the mechanism of how *SATB2* mutation results in tooth agenesis in human is rarely studied.

Human dental pulp stem cells (hDPSCs) are a population of mesenchymal stem cells which harbor the characteristics of rapid proliferation, self-renewal capacity, and multilineage differentiation potential [11, 12]. Tooth morphogenesis is regulated by interactions between dental epithelial and mesenchyme thus stimulating hDPSCs to differentiate into odontoblasts, which form primary dentin. The timely and precise regulation of proliferation and differentiation of hDPSCs under various molecular networks is crucial for odontogenesis and normal tooth pattern [13–15]. In one of our previous studies, we identified a *MSX1* mutation in patients with familial nonsyndromic tooth agenesis and confirmed that the mutation could downregulate odontogenic differentiation capacity of hDPSCs via the ERK pathway [16]. However, the roles of plenty of other odontogenic genes which have been reported to be associated with tooth agenesis such as *SATB2, BMP4, GREMLIN2, LRP6* and the mechanism of decreased function of hDPSCs in patients with tooth agenesis is still not fully understood [3, 17–19].

Epigenetic modulation plays a vital role in tissue and organ development and disease progression. Histone modification is one of the most common form of epigenetic modulation [20, 21]. Epigenetic therapies such as histone deacetylase inhibitors have been proved partially effective in some diseases, such as cancer and neurologic conditions [22]. Epigenetic factors also have indispensable effects during odontogenesis and tooth development [23]. Mutation of genes causing tooth agenesis might alter the epigenetic status of downstream genes, thus affecting odontogenesis of dental mesenchymal stem cells and tooth development. An increasing number of researches have proved that epigenetic modulation could regulate the proliferation and differentiation function of stem cells, which shed light on novel therapies of stem cell-based-tissue regeneration [24]. The underlying epigenetic mechanism such as histone modification regulating function of dental mesenchymal stem cells and tooth development remains to be further investigated.

In this study, we identified a novel frameshift mutation of *SATB2* in a Chinese patient diagnosed as *SATB2*-associated syndrome who showed permanent teeth congenitally missed. Mechanistically, the mutation of *SATB2* affects the odontogenic differentiation capacity of hDPSCs through Wnt/*β*-catenin signaling pathway by regulating DKK1 and histone demethylase JHDM1D.

## Material and methods

### Clinical diagnosis and sample collection

The patient and her parents came to the Department of Pediatrics in Peking University First Hospital because of developmental delay and limited speech. Clinical manifestation, past medical history, family history, video electroencephalography (EEG), magnetic resonance imaging (MRI) and genetic data of the patient were acquired. Then she was referred to Peking University School and Hospital of Stomatology to have oral examination due to hypodontia and tooth malformation. Facial and intraoral photos of the patient were taken while radiograph was not obtained due to her noncompliance. This study was approved by the Ethics Committee of Peking University First Hospital (2012453) and Ethics Committee of Peking University School and Hospital of Stomatology (2013053). Written informed consent for publication of the patient’s clinical details and images was obtained from the parents of the patient.

### Genetic analysis and construction of expression vectors

Genomic DNA of the patient and her parents was extracted from peripheral blood using the QIAmp Blood minikit (Qiagen, Venlo, the Netherlands). Whole-exome sequencing (WES) was applied to detect mutation. All samples were sequenced on an Illumina platform by Euler Genomics (Beijing, China). Synonymous changes and single nucleotide polymorphisms with minor allele frequency (MAF) higher than 5% were removed. Clinical significance and pathogenicity of the identified variant was interpreted according to the guidelines set by the American College of Medical Genetics (ACMG) [25], and was further validated by Sanger sequencing. Three-dimensional model of the variant compared with the wild-type was predicted by Swiss model. The human SATB2 cDNA and the mutant SATB2 cDNA with Flag-tag were cloned into pcDNA3.1 expression vector respectively.

### Cell culture

Human dental pulp stem cells (hDPSCs) were isolated from healthy dental pulp tissue of orthodontic extracted premolars or healthy third molars, and cultured as we previously reported [16]. The protocol to acquire human dental pulp tissue was approved by the Ethics Committee of Peking University School and Hospital of Stomatology (PKUSSIRB-201311103).

### Flow cytometry analysis

Fluorescently conjugated antibodies including anti-human CD29-PE, CD73-PE, CD34-APC, CD45-APC (BD Biosciences, USA) were used for cell surface marker staining. The data were acquired and analyzed by a BD Accuri C6 flow cytometer platform (BD Biosciences, USA).

### Transient transfection

We purchased SATB2 and JHDM1D small interfering RNA (siRNA) oligonucleotides from GenePharma (Suzhou, China). The sequences were as follows: 5′-GCUUAGUCCACAACUUGUAdtdt-3′ (SATB2 sense), 5′-UACAAGUUGUGGACUAAGCdtdt-3′ (SATB2 anti-sense), 5′-CAAGUGCCGAUGAAAUAAUtt-3′ (JHDM1D sense), 5′-AUUAUUUCAUCGGCACUUGtt-3′ (JHDM1D anti-sense). Lipofectamine RNAiMAX Reagent (Invitrogen, USA) was used to transfect siRNA into hDPSCs. Wild-type or mutant Flag-SATB2 expression vectors were transfected into hDPSCs with lipofectamine 3000 Reagent (Invitrogen, USA). After 24–48 h, cells were harvested for analysis or cultured for follow-up experiments.

### Fluorescent immunocytochemistry

Cells were fixed in 4% paraformaldehyde first, then permeabilized with 0.1% Triton X-100 and blocked with 5% bovine serum albumin (Solarbio, China). Anti-Flag (1:1000; Origene, USA), anti-SATB2 (1:10; Abcam, USA) and anti-BrdU (1:1000; Invitrogen, USA) were used for overnight incubation at 4℃, and fluorescent secondary antibody (red) was used for 1 h incubation at room temperature. At last, the slides were mounted with mounting media containing DAPI (blue) to stain the nuclei before observed with a laser scanning confocal microscope (Zeiss, Germany). For BrdU assay, three independent samples were used for each group. Cell numbers of BrdU-positive cells and total cells were counted in 5 images per sample.

### Western blotting

RIPA buffer mixed with protease inhibitor (Thermo Fisher Scientific, USA) was used to lyse cells for harvest of total protein. The protein was separated by 4–20% Precast-Gel (Solarbio, China) and transferred to a PVDF membrane (Millipore, USA). Then 5% bovine serum albumin was used to block the membrane for 1 h at room temperature, and incubated with primary antibody overnight at 4℃. The following primary antibodies were used: anti-Flag (Origene, USA), anti-SATB2 (Abcam, USA), anti-*β*-catenin, anti-active* β*-catenin (Cell Signaling Technology, USA), anti-*β*-actin (Zhongshanjinqiao, China), anti-RUNX2 (Biorbyt, England), anti-JHDM1D (Abcam, USA), anti-DKK1 (Santa Cruz, USA), anti-H3K9me2, anti-H3K27me2 (Abcam, USA). HRP-conjugated secondary antibody (1:10,000; Zhongshanjinqiao, China) was then used to incubate the membrane for 1 h at room temperature. At last, we used ECL and Super Signal detection reagents (Thermo Fisher Scientific, USA) to detect the membrane.

### Quantitative real-time PCR

TRIzol reagent (Thermo Fisher Scientific, USA) was used to extract total RNA of cells. A PrimeScript RT Master Mix system (Takara, Japan) was used to synthesize cDNA. Real-time PCR was performed using SYBR Master Mix (Roche, Switzerland). Primers for real-time PCR are listed in Additional file 1: Table S1.

### Alizarin Red S staining

After 14 days of culturing under odontogenic medium, hDPSCs were fixed using 4% paraformaldehyde and stained with 1% alizarin red S staining solution (Sigma, USA) for 1 min at room temperature, following manufacturer’s instruction. After washed with deionized water for several times, cells were visualized under a microscope to analyze calcium nodule formation. Quantification of calcium deposition was conducted by Image J. Three views under microscope were chosen randomly in each group, and the area stained by alizarin red S staining solution was calculated by Image J software. The composition of odontogenic medium was the same as we described previously [16].

### Statistical analysis

Each experiment was repeated for three times at least. SPSS Statistics 20 software was applied for analysis. Student’s *t* test and one-way analysis of variance (ANOVA) were used for comparison between two groups and more than two groups, respectively.

## Results

### Clinical characteristics of the patient

**Fig. 1:**
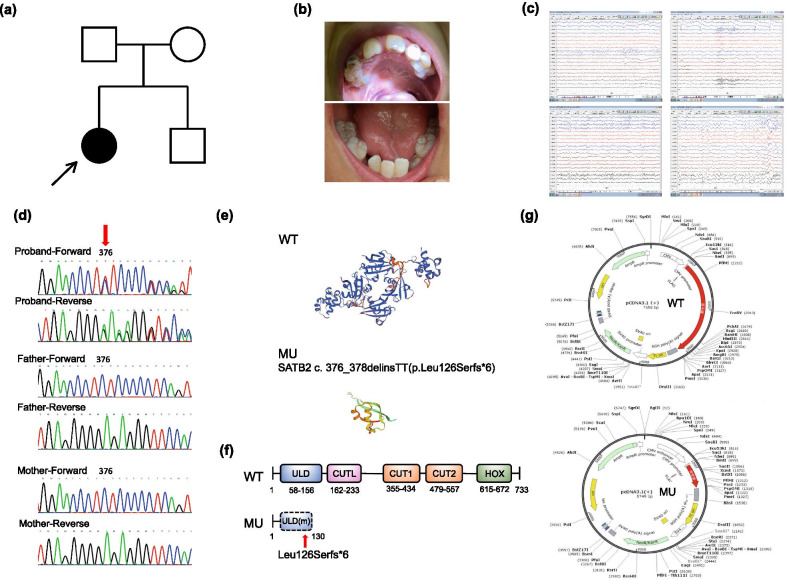
Clinical information and mutation analysis of the SAS patient and her parents. **a** Pedigree of the patient’s family. Arrow indicates the patient. The patient’s parents and brother are all healthy individuals. **b** Intraoral photos of the patient. Several permanent teeth are congenitally missing. **c** Abnormal electroencephalogram(EEG) of the patient. **d** Sanger chromatograms of the patient and her parents. A novel frameshift mutation of *SATB2* (c. 376_378delinsTT) was identified in the patient. The genotype of her parents is normal. **e** Three-dimensional models of wild-type and mutant SATB2. **f** Schematic diagram of wild-type and mutant SATB2 protein with domains according to Pfam database (http://pfam.xfam.org/). The mutant protein synthesis was blocked at the ubiquitin-like oligomerization domain (ULD) **g** Construction of wild-type and mutant SATB2 expression vectors

The patient was 12 years old when she first visited the Department of Pediatrics in Peking University First Hospital. She had been through operation of cleft palate when she was 3 years old. The patient exhibited developmental delay, growth retardation and speech disorder. The patient’s parents and brother were all healthy individuals (Fig. 1a). Intraoral photos showed abnormal dentition with hypodontia and crowding (Fig. 1b). Interracial electroencephalogram showed bilateral asymmetric rhythm and paroxysmal activities (Fig. 1c).

### Mutation of SATB2 and expression vectors

Whole exome sequencing revealed a novel frameshift mutation of *SATB2* (c. 376_378delinsTT) in the patient, while no pathogenic mutation was identified in her parents. Sanger sequencing was applied to verify the identified *SATB2* mutation in the patient and her parents’ genotype were normal (Fig. 1d). Three-dimensional structure of wild-type and mutant SATB2 was constructed using Swiss model, which demonstrated a significant structure change of the mutant protein (Fig. 1e). Schematic diagram of wild-type and mutant SATB2 protein with domains was presented according to Pfam database (http://pfam.xfam.org/), and the mutant protein synthesis was blocked at the ubiquitin-like oligomerization domain (ULD) (Fig. 1f). Wild-type and mutant *SATB2* cDNA with Flag-tag was cloned into pcDNA3.1, respectively, to generate wild-type and mutant SATB2 expression vector (Fig. 1g).

### SATB2 mutation affected nuclear localization of SATB2 protein in hDPSCs

**Fig. 2:**
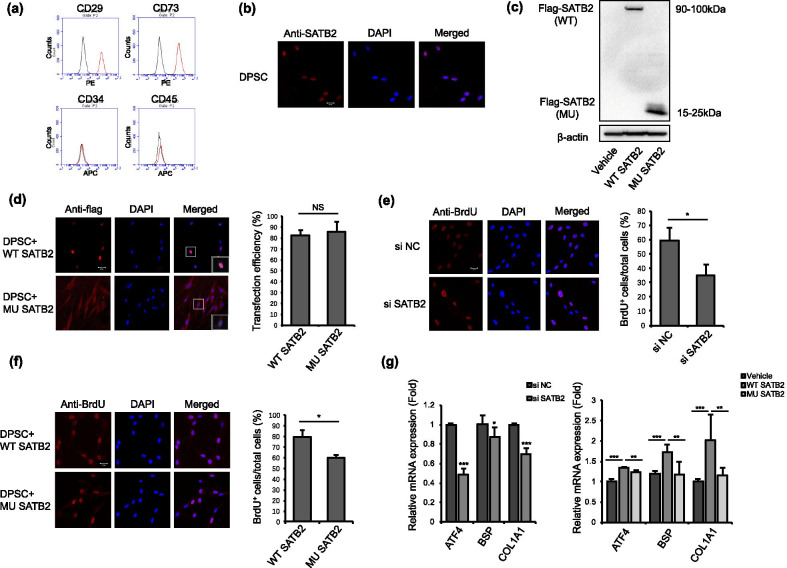
Identification of human dental pulp stem cells and influence of SATB2 on proliferation rate of hDPSCs. **a** Flow cytometry assays showed that hDPSCs were positive for CD29 and CD73, and negative for CD34 and CD45. **b** Cell immunofluorescence showed that intrinsic SATB2 expressed exclusively in the nucleus. **c** Western blot analysis showed that the molecular weight of mutant SATB2 was significantly lower than the wild-type. **d** Cell immunofluorescence of transfected flag-tagged SATB2 in hDPSCs. Wild-type SATB2 located in the nucleus while mutant SATB2 was distributed over the cytoplasm. Transfection efficiency of both wild-type and mutant SATB2 was around 80%. **e** BrdU-labeling assay showed that the proliferation rate of hDPSCs transfected with SATB2 siRNA was lower than negative control. **f** BrdU-labeling assay showed that the proliferation rate of hDPSCs transfected with mutant SATB2 was lower than cells transfected with wild-type. **g** SATB2 knockdown in hDPSCs resulted in decreased expression of ATF4, BSP and COL1A1. Transfection of wild-type SATB2 upregulated the expression of ATF4, BSP and COL1A1 compared with vehicle, while cells transfected with mutant SATB2 expressed lower level of ATF4, BSP and COL1A1 compared with wild-type. Data are expressed as the means + SD. Each experiment was repeated three times with n ≥ 3 samples per group. Scale bar 20 μm, *P < 0.05, ** *P* < 0.01, *** *P* < 0.001, NS non-significant. si NC: negative control, siRNA transfection, si SATB2: SATB2 siRNA transfection, Vehicle: vector transfection, WT SATB2: wild-type SATB2 transfection, MU SATB2: mutant SATB2 transfection

We analyzed the characteristic of hDPSCs and found that hDPSCs were positive for mesenchymal stem cell surface marker CD73 and CD 29, negative for hematopoietic stem cell marker CD45 and CD34 (Fig. 2a). Immunostaining results showed that SATB2 was expressed in hDPSCs and localized in the nucleus of hDPSC exclusively (Fig. 2b). To find out whether the mutation affected nuclear localization of SATB2, we transfected wild-type and mutant SATB2 vector in hDPSCs and found that both the transfected wild-type and mutant SATB2 expressed in hDPSCs while the molecular weight of mutant SATB2 was significantly lower (Fig. 2c). Moreover, the wild-type SATB2 localized in the nucleus while mutant SATB2 localized in the cytoplasm (Fig. 2d). These results indicated that mutant SATB2 failed to translocate into nucleus.

### The mutation of SATB2 decreased proliferation rate of hDPSCs

To investigate the influence of SATB2 on cell proliferation, we used SATB2 siRNA to knock down SATB2 expression in hDPSCs and found that the percentage of BrdU-positive cells was decreased compared with control ones (Fig. 2e). To analyze the impact of mutant SATB2 on proliferation rate of hDPSCs, the percentage of BrdU-positive cells was calculated after transfected with wild-type and mutant SATB2. DPSCs transfected with mutant SATB2 showed significantly lower percentage of BrdU-positive cells compared with wild-type (Fig. 2f). We also conducted live/dead viability assay after transfection, and the results demonstrated that both wild-type and mutant SATB2 decreased cell viability compared with control, but no significant difference was found between wild-type and mutant groups (Additional file 1: Fig. S1). It was previously reported that SATB2 could target downstream genes like ATF4, BSP, and COL1A1 [5, 26]. After knockdown of SATB2 by siRNA, the expression of ATF4, BSP and COL1A1 was significantly decreased. The expression of ATF4, BSP and COL1A1 was significantly increased after WT SATB2 transfection in hDPSCs compared with vehicle, while the expression level of ATF4, BSP and COL1A1 was significantly lower in mutant SATB2 transfection group compared to the wild-type ones (Fig. 2g).

### SATB2 regulated osteo/odontogenic differentiation of hDPSCs

**Fig. 3:**
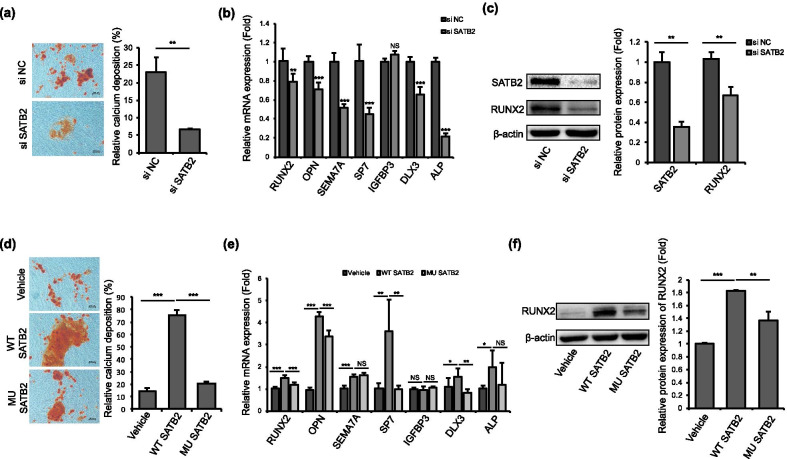
Effects of SATB2 on osteo/odontogenic differentiation of hDPSCs. **a** Calcium nodule deposition of hDPSCs examined by Alizarin Red S staining showed that SATB2 knockdown significantly decreased the capacity of osteo/odontogenic differentiation of hDPSCs compared with the control group. **b** Quantitative RT-PCR revealed that the mRNA level of RUNX2, OPN, SEMA7A, SP7, DLX and ALP, but not IGFBP3, decreased after knockdown of SATB2. **c** Western blot analysis showed that RUNX2 was significantly downregulated after SATB2 knockdown compared with the control ones. **d** The DPSCs transfected with wild-type SATB2 showed increased capacity of osteo/odontogenic differentiation compared to vehicle, while cells transfected with mutant SATB2 showed decreased capacity of osteo/odontogenic differentiation compared to the ones transfected with wild-type SATB2. **e** Transfection of wild-type SATB2 upregulated the expression of RUNX2, OPN, SEMA7A, SP7, DLX and ALP, but not IGFBP3, compared with vehicle. Cells transfected with mutant SATB2 expressed lower level of RUNX2, OPN, SP7 and DLX3 compared with wild-type. **f** Western blot analysis showed that RUNX2 was up-regulated when hDPSCs were transfected with wild-type SATB2, while mutant SATB2 transfection showed lower expression level of RUNX2 compared with the wild-type. Each experiment was repeated three times with n ≥ 3 samples per group. Scale bar 200 μm, *P < 0.05, **P < 0.01, ***P < 0.001. Data are expressed as the means + SD. si NC: negative control siRNA transfection, si SATB2: SATB2 siRNA transfection, Vehicle: vector transfection, WT SATB2: wild-type SATB2 transfection, MU SATB2: mutant SATB2 transfection

We transfected SATB2 siRNA into hDPSCs and induced osteo/odontogenic differentiation. The results demonstrated that SATB2 knockdown significantly decreased the odontogenic differentiation potential of hDPSCs compared with control group (Fig. 3a). The expression of osteo/odontogenic differentiation related marker RUNX2, OPN, SEMA7A, SP7, DLX3 and ALP, but not IGFBP3, was significantly downregulated after SATB2 knockdown (Fig. 3b, c).

Furthermore, we transfected wild-type and mutant SATB2 to hDPSCs and the results showed that hDPSCs transfected with mutant SATB2 showed a decreased calcium nodule formation ability compared with wild-type ones (Fig. 3d). Transfection of wild-type SATB2 upregulated the expression of RUNX2, OPN, SEMA7A, SP7, DLX and ALP, but not IGFBP3, compared with vehicle. Cells transfected with mutant SATB2 expressed lower level of RUNX2, OPN, SP7 and DLX3 compared with wild-type one. (Fig. 3e, f).

### SATB2 regulates odontogenesis of hDPSCs through Wnt/*β*-catenin pathway

**Fig. 4:**
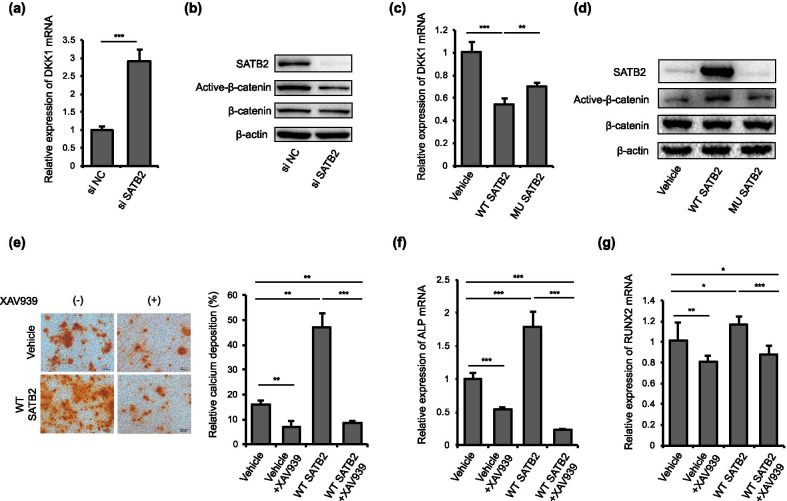
SATB2 regulated osteo/odontogenic differentiation of hDPSCs through Wnt/*β*-catenin signaling pathway. **a** Quantitative RT-PCR showed that SATB2 knockdown remarkably up-regulated DKK1 expression. **b** Western blot analysis showed that SATB2 knockdown down-regulated active* β*-catenin expression. **c** The hDPSCs transfected with wild-type SATB2 showed lower level of DKK1 compared with vehicle, while mutant SATB2 transfection expressed higher level of DKK1 compared with the ones transfected with wild-type SATB2, as assessed by q-PCR analysis. **d** Wild-type SATB2 transfection induced higher level of active* β*-catenin compared with vehicle. The expression of active* β*-catenin was lower in the hDPSCs transfected with mutant SATB2 in comparison to those transfected with wild-type SATB2. **e** Alizarin Red S staining showed that XAV939 decreased osteo/odontogenesis of hDPSCs and the increased mineralized nodule formation induced by SATB2 transfection was blocked by XAV939. **f**, **g** Quantitative RT-PCR showed that XAV939 decreased ALP and RUNX2 expression in hDPSCs. The increased expression of ALP and RUNX2 induced by wild-type SATB2 transfection was impaired after the inhibition of Wnt/*β*-catenin signaling pathway. Scale bar 200 μm, *P < 0.05, **P < 0.01, ***P < 0.001. Data are expressed as the means + SD. Each experiment was repeated three times with n ≥ 3 samples per group. si NC: negative control siRNA transfection, si SATB2: SATB2 siRNA transfection, Vector: vector transfection, WT SATB2: wild-type SATB2 transfection, MU SATB2: mutant SATB2 transfection, Vector + XAV939: vector transfection and XAV939 added, WT + XAV939: wild-type SATB2 transfection and XAV939 added

Wnt/*β*-catenin pathway has been proved to play a critical role in odontogenic differentiation and dentin formation [27, 28]. DKK1 is largely known as an inhibitor of Wnt/*β*-catenin pathway [29]. We found that DKK1 expression was upregulated after SATB2 knockdown (Fig. 4a). The level of active* β*-catenin was also significantly decreased after knockdown of SATB2 in comparison with control group (Fig. 4b). Moreover, the level of DKK1 was higher in hDPSCs transfected with mutant SATB2 compared with those transfected with wild-type SATB2 (Fig. 4c). The expression level of active* β*-catenin was decreased in hDPSCs transfected with mutant SATB2 compared with those transfected with wild-type SATB2 (Fig. 4d). To further investigate the role of Wnt/*β*-catenin signaling pathway in SATB2-induced osteo/odontogenic differentiation of hDPSCs, XAV939 (Wnt inhibitor) was used to inhibit Wnt/*β*-catenin signaling pathway after transfection of vehicle or wild-type SATB2. Alizarin Red S staining showed that XAV939 inhibited calcium nodule formation capacity of hDPSCs, and the increased osteo/odontogenic differentiation of hDPSCs induced by SATB2 overexpression was attenuated after XAV939 treatment (Fig. 4e). The expression of ALP and RUNX2 was also decreased after XAV939 treatment both in vehicle and SATB2 transfection groups (Fig. 4f, g).

### Histone demethylase JHDM1D is associated with SATB2-mediated osteo/odontogenic differentiation of hDPSCs

**Fig. 5:**
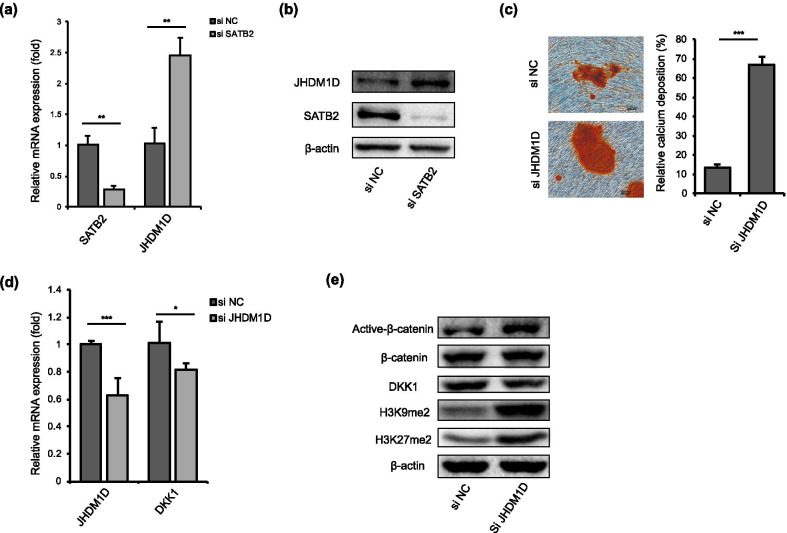
Histone demethylase JHDM1D is associated with SATB2-mediated osteo/odontogenic differentiation of hDPSCs. **a**, **b** The expression of histone demethylase JHDM1D was increased after SATB2 knockdown, as assessed by quantitative RT-PCR and western blot analysis. **c** Alizarin Red S staining showed that JHDM1D knockdown upregulated mineralized nodule formation capacity of hDPSCs. **d** Quantitative RT-PCR showed that DKK1 expression was down-regulated after JHDM1D knockdown. **e** Western blot analysis showed that JHDM1D knockdown upregulated the level of H3K9me2 and H3K27me2, and increased the expression of active* β*-catenin. Scale bar 200 μm, *P < 0.05, **P < 0.01, ***P < 0.001. Data are expressed as the means + SD. Each experiment was repeated three times with n ≥ 3 samples per group. si NC: negative control siRNA transfection, si SATB2: SATB2 siRNA transfection, si JHDM1D: JHDM1D siRNA transfection

JHDM1D is a histone lysine demethylase which has been reported to inhibit osteogenesis of bone marrow mesenchymal stem cells (BMSCs) [30, 31]. To explore whether JHDM1D is involved in SATB2-mediated osteo/odontogenesis of hDPSCs, we transfected SATB2 siRNA and found that the expression of JHDM1D was upregulated after SATB2 knockdown (Fig. 5a, b). The mineralized nodule formation of hDPSCs was increased after JHDM1D knockdown by siRNA treatment (Fig. 5c). Furthermore, DKK1 expression was downregulated while active* β*-catenin expression was upregulated after JHDM1D knockdown (Fig. 5d, e). The level of H3K9me2 and H3K27me2 was also upregulated after JHDM1D was inhibited (Fig. 5e).

## Discussion

We identified a novel frameshift mutation (c.376_378delinsTT, p.Leu126SerfsX6) of *SATB2* in a patient diagnosed as *SATB2*-associated syndrome (SAS). The patient’s clinical manifestation including hypodontia, cleft palate, dysmorphic facial features, developmental delay and abnormal electroencephalography, was consistent with those reported in the literature [3, 10, 32, 33]. Up till now, more than 120 different types of pathogenic mutation of human *SATB2* were identified, leading to defects in several organs [2, 34]. SATB2 is a transcription factor which binds to matrix-attachment regions (MARs) and plays a crucial role in different developmental process [4–6, 35]. In human, SATB2 is expressed in dental papilla cells, odontoblasts, dental pulp cells, and involved in the dentin mineralization process [36, 37]. It has been reported that among all types of dental mesenchymal stem cells, human dental pulp stem cells (hDPSCs) exhibit the highest expression of SATB2 [38]. However, how *SATB2* mutation results in human dental anomaly, and its impact to the function of hDPSCs and the underlying mechanism is rarely reported. Here we showed that the mutation of SATB2 interrupted its nuclear localization and decreased proliferation rate of hDPSCs. Todd et al. have revealed that mutated *Satb2* resulted in reduced proliferation capacity of mice pre-osteoblast [39]. As SATB2 is an MARs-binding protein, mutation of *SATB2* may disrupt its ability to regulate cell-cycle related genes or have an impact on DNA replication, thus leading to reduction in proliferation rate of hDPSCs. The exact mechanism of SATB2-regulated cell proliferation needs to be further investigated in the future.

SATB2 is expressed during mice molar development, and *Satb2*^−/−^ osteoblast differentiation function was significantly reduced compared with wild-type [5]. We found that SATB2 knockdown significantly decreased odontogenic capacity of hDPSCs, and wild-type SATB2 transfection remarkably increased odontogenic capacity of hDPSCs compared with vehicle ones. Mutant SATB2 decreased odontogenic differentiation of hDPSCs in comparison with wild-type SATB2. These results indicated that the novel variant of SATB2 we identified in this SAS patient may downregulate the mineralized tissue formation capacity of her dental pulp stem cells, partially leading to the phenotype of tooth agenesis in this patient. Dong et al. reported that SATB2 could promote osteogenic differentiation of BMMSCs [40]. Our results confirmed that mutation of SATB2 decreased osteo/odontogenic differentiation of hDPSCs, and is important for normal teeth development in human. Taken together, SATB2 plays a critical role on hard tissue formation and is a potential target for modulating osteogenic differentiation properties of human mesenchymal stem cells.

Dobreva et al. reported that SATB2 directly interacts with and enhances the activity of RUNX2. SATB2 was also found to repress the expression of HOXA2, an inhibitor of bone formation [5]. Hassan et al. have reported that RUNX2 negatively regulated multiple miRNAs by a feed-forward mechanism to cause depression of SATB2 in murine preosteoblast cell line MC3T3 cells [41]. Hu et al. found that SATB2 could rescue the negative effect of mir-205 on osteogenesis via activating the expression of RUNX2 in BMSCs [42]. The precise role of SATB2 regulating RUNX2 may be context dependent and needs further investigation to illustrate how they interact in hDPSCs.

The effects of SATB2 mutation seem not so significant compared to the SATB2 siRNA treatment, which may be due to the N-terminal ubiquitin-like domain (ULD) residue. Wang et al. have reported that ULD-mediated oligomerization is required for binding to target DNA, since its dimer or tetramer may regulate gene expression by recognizing specific DNA sequences in the promoter regions of various genes [43]. Mutant SATB2 might still be able to regulate genes through DNA–protein or protein–protein interaction with its remaining DNA sequence or protein domain. Further study is needed to fully illustrate the mechanism of how mutant SATB2 regulates downstream genes in hDPSCs.

Furthermore, our results showed that SATB2 knockdown upregulated the level of DKK1 while downregulated active* β*-catenin expression. When transfected with mutant SATB2, DKK1 expression was increased and active* β*-catenin expression was decreased compared with the wild-type. To further analyze the role of Wnt/*β*-catenin signaling pathway in SATB2-induced odontogenesis of hDPSCs, Wnt inhibitor XAV939 was used to treat cells after transfection of wild-type SATB2. The enhanced odontogenic differentiation capacity of hDPSCs induced by SATB2 was blocked by Wnt inhibitor XAV939. These results revealed that SATB2 regulates odontogenesis of hDPSCs via the Wnt/*β*-catenin signaling pathway.

Epigenetic regulation on function of mesenchymal stem cells is attracting more and more attention these years [44, 45]. JHDM1D, also known as KDM7A, is a histone demethylase specific for removing di-methylation marks on histone H3 lysine 9 and lysine 27 on promoters of target genes [46, 47]. Yang et al. have reported that JHDM1D might be able to inhibit osteogenesis of ST2 cells (mice bone marrow cells) by inactivating Wnt signaling pathway [31]. To investigate whether JHDM1D regulated SATB2-Wnt/*β*-catenin-involved odontogenesis of hDPSC, we detected the expression of JHDM1D after SATB2 siRNA transfection. The results showed that the expression of JHDM1D was significantly upregulated after SATB2 knockdown. JHDM1D knockdown enhanced mineralized nodule formation capacity of hDPSCs and upregulated the expression of active* β*-catenin by downregulating DKK1. These results demonstrate, for the first time, that SATB2 is capable of regulating odontogenesis of hDPSCs through activating Wnt/*β*-catenin signaling pathway by inhibiting JHDM1D expression. Epigenetic modification of DKK1 has been proved to act on Wnt/*β*-catenin signaling pathway, such as DNA demethylation mediated by TET1 during development and progression of cancer [48, 49]. Also, DKK1 is reported to be an important target of histone demethylase KDM6A/KDM7A that mediates endoderm differentiation of human ESCs [50]. Based on the previous studies and our findings, mutation of SATB2 could upregulate histone demethylase JHDM1D, and histone modification of DKK1 by JHDM1D might regulate Wnt/*β*-catenin signaling pathway thus mediating osteo/odontogenic differentiation of hDPSCs. To the best of our knowledge, the relationship between DKK1 and JHDM1D has not been reported before. Whether JHDM1D could directly regulate histone modification of DKK1 or through other targeted genes thus affecting osteo/odontogenic differentiation may need to be further investigated.

Our finding provides new pathogenic mechanism for SAS patients with tooth agenesis, which may be resulted from reduced capacity of proliferation and odontogenic differentiation of hDPSCs. Epigenetic modulation also plays a role in this developmental disorder. Function enhancement of hDPSCs by activating Wnt/*β*-catenin signaling pathway or through JHDM1D-induced epigenetic modulation may be considered as new therapeutic strategies for SAS patients with the manifestation of tooth agenesis. Since SAS patients exhibit defects of multiple organs, current treatment strategies are mainly symptom-guided [10, 33, 51, 52]. Underlying cellular and molecular pathogenic mechanism of SAS needs to be further investigated. Epigenetic modulation including histone modification might be a promising approach treating SAS in the future.

## Conclusion

**Fig. 6:**
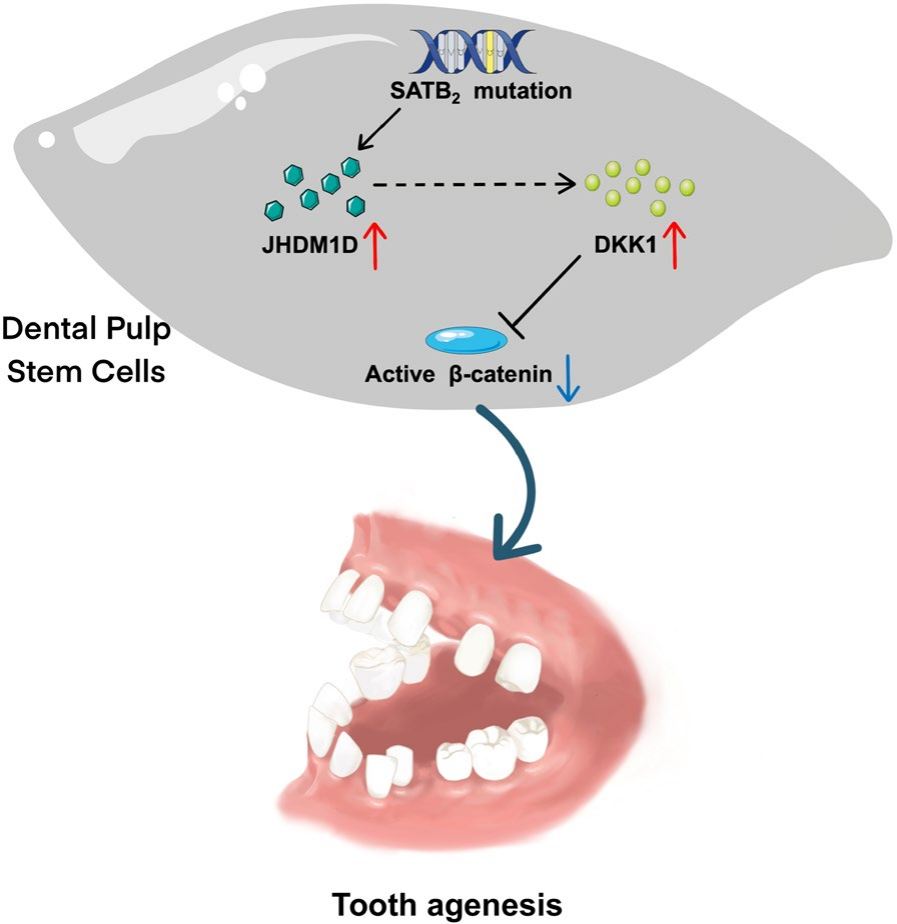
Schematic showing SATB2 mutation inhibits odontogenesis of hDPSCs through Wnt/*β*-catenin signaling pathway by regulating DKK1 and histone demethylase JHDM1D, thus leading to the phenotype of tooth agenesis

In summary, we identified a novel frameshift mutation of SATB2 (c.376_378delinsTT, p.Leu126SerfsX6) in a Chinese patient with *SATB2*-associated syndrome (SAS) exhibiting tooth agenesis. This novel SATB2 variant inhibited osteo/odontogenesis of hDPSCs through Wnt/*β*-catenin signaling pathway by regulating DKK1 and histone demethylase JHDM1D, thus leading to the phenotype of tooth agenesis in the SAS patient (Fig. 6). This underlying mechanism shed new light on pathogenesis of SAS, which will provide new clues for future therapeutic strategies for this disease.

## Supplementary Information

**Additional file 1**.** Supplementary Figure 1.** Living/dead viability assay of cells after transfection of wild-type and mutant SATB2. Cells stained with calcein AM in green manifested living hDPSCS while cells stained with ethidium homodimer-1 (EthD-1) in red were dead. Both wild-type and mutant SATB2 decreased cell viability compared with control, but no significant difference was found between wild-type and mutant. **P < 0.01, NS non-significant. Data are expressed as the means+SD. Each experiment was repeated three times with n ≥ 3 samples per group.** Supplementary Table 1.** Primer list for quantitative real-time PCR.

## Data Availability

The authors confirm that all data generated or analyzed during this study are available.

## Abbreviations

SATB2: Special AT-rich sequence binding protein 2
SAS: *SATB2*-Associated syndrome
hDPSCs: Human dental pulp stem cells
WES: Whole exome sequencing
JHDM1D: Histone demethylase 1 homolog D
DAPI: 4′,6′-Diamidino-2-phenylindole
ARS: Alizarin Red S
RUNX2: RUNX family transcription factor 2
ALP: Alkaline phosphatase
DKK1: Dickkopf WNT signaling pathway inhibitor 1
