# Supplementary material for: A novel mutation of SATB2 inhibits odontogenesis of human dental pulp stem cells through Wnt/β-catenin signaling pathway
Source: Stem Cell Res Ther. 2021 Dec 4;12:595. doi: 10.1186/s13287-021-02660-8 (PMC8642962; doi:10.1186/s13287-021-02660-8)
Supplement: Supplementary file 1 — Additional file 1. Supplementary Figure 1. Living/dead viability assay of cells after transfection of wild-type and mutant SATB2. Cells stained with calcein AM in green manifested living hDPSCS while cells stained with ethidium homodimer-1 (EthD-1) in red were dead. Both wild-type and mutant SATB2 decreased cell viability compared with control, but no significant difference was found between wild-type and mutant. **P < 0.01, NS non-significant. Data are expressed as the means+SD. Each experiment was repeated three times with n ≥ 3 samples per group. Supplementary Table 1. Primer list for quantitative real-time PCR. [file 13287_2021_2660_MOESM1_ESM.pdf]

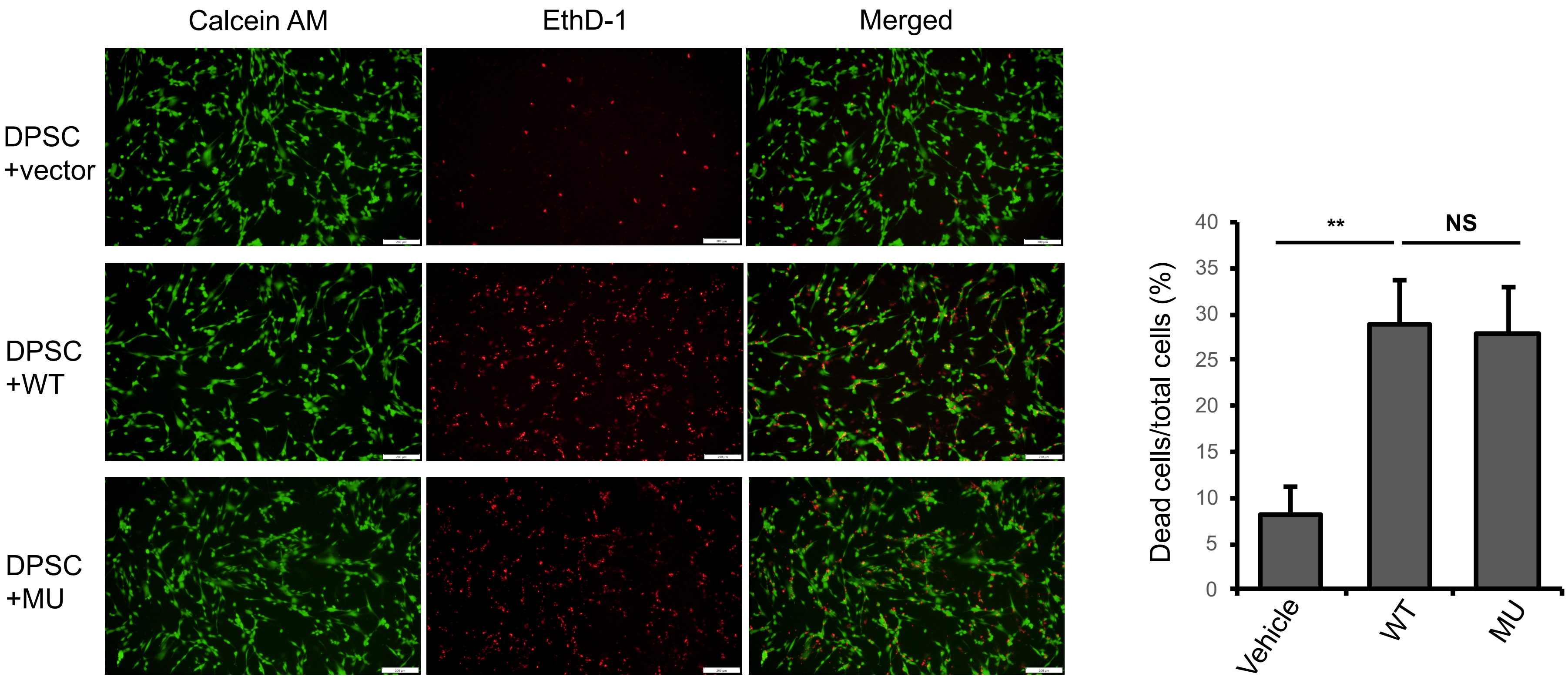


**Supplementary Figure. 1** Living/dead viability assay of cells after transfection of wild-type and mutant SATB2. Cells stained with calcein AM in green manifested living hDPSCS while cells stained with ethidium homodimer-1 (EthD-1) in red were dead. Both wild-type and mutant SATB2 decreased cell viability compared with control, but no significant difference was found between wild-type and mutant. ** $P$<0.01, NS non-significant. Data are expressed as the means+SD. Each experiment was repeated three times with n≥3 samples per group.

**Supplementary Figure 1**

| Gene | Forward primer (5' to 3') | Reverse primer (5' to 3') |
|---|---|---|
| SATB2 | GGAGAACGACAGCGAGGAA | CCGATGTATTGCTTTGCCTAGT |
| GAPDH | GGAGCGAGATCCCTCCAAAAT | GGCTGTTGTCATACTTCTCATGG |
| RUNX2 | CCGCCTCAGTGATTTAGGGC | GGGTCTGTAATCTGACTCTGTCC |
| ALP | AACATCAGGGACATTGACGTG | GTATCTGGTTTGAAGCTCTTCC |
| DKK1 | CCTTGAACTCGGTTCTCAATTCC | CAATGGTCTGGTACTTATTCCCG |
| JHDM1D | GTGGAGGTCCCTGATATAGCC | CCACCGAAGTCAATGTGGAAA |
| ATF4 | CTCCGGGACAGATTGGATGTT | GGCTGCTTATTAGTCTCCTGGAC |
| SEMA7A | CACCAAGACCAGGCTTACGAT | ACACGGGACACATTGAGAGGA |
| COL1A1 | GAGGGCCAAGACGAAGACATC | CAGATCACGTCATCGCACAAC |
| IGFBP3 | AGAGCACAGATACCCAGAACT | GGTGATTCAGTGTGTCTTCCATT |
| DLX3 | CTCGCCCAAGTCGGAATATAC | CTGGTAGCTGGAGTAGATCGT |
| BSP | AAAGTGAGAACGGGGAACCT | GATGCAAAGCCAGAATGGAT |
| OPN | GAAGTTTCGCAGACCTGACAT | GTATGCACCATTCAACTCCTCG |
| SP7 | GAGGCAACTGGCTAGGTGG | CTGGATTAAGGGGAGCAAAGTC |

**Supplementary Table. 1** Primer list for quantitative real-time PCR.
